# Supplementary material for: Do B Vitamins Enhance the Effect of Omega-3 Polyunsaturated Fatty Acids on Cardiovascular Diseases? A Systematic Review of Clinical Trials
Source: Nutrients. 2022 Apr 12;14(8):1608. doi: 10.3390/nu14081608 (PMC9032763; doi:10.3390/nu14081608)
Supplement: Supplementary file 1 [file nutrients-14-01608-s001.zip › nutrients-1637059-supplementary.pdf]

Table S1. Research validity assessment using the American Dietetic Association's Quality Criteria Checklist.

Reference	1. Clear statement of research question	2. Participant selection free from bias	3. Comparable study groups	4. Response rate or withdrawals described	5. Use of blinding	6. Intervention protocol and/or data collection procedure described in detail	7. Outcomes clearly defined and the measurements valid and reliable	8. Appropriate statistical analysis	9. Conclusion supported by results	10. Unlikely funding or sponsorship bias	Overall quality rating *
Haglund et al. (1993), Sweden [12]	Yes	No	Yes	Yes	Yes	Yes	Yes	Yes	Yes	Unclear	Ø
Baró et al. (2003), Spain [24]	Yes	Yes	Yes	Yes	No	Yes	Yes	Yes	Yes	Unclear	+
Carrero et al. (2004), Spain [25]	Yes	Yes	Yes	Yes	No	Yes	Yes	Yes	Yes	Unclear	+
Carrero et al. (2005), Spain [20]	Yes	No	Yes	Yes	Yes	Yes	Yes	Yes	Yes	Unclear	Ø
Benito et al. (2006), Spain [21]	Yes	Yes	Yes	Yes	No	Yes	Yes	Yes	Yes	Unclear	+
Carrero et al. (2006), Spain [23]	Yes	No	Yes	Yes	Yes	Yes	Yes	Yes	Yes	Unclear	Ø
Carrero et al. (2007), Spain [22]	Yes	No	Yes	Yes	Yes	Yes	Yes	Yes	Yes	Unclear	Ø
Fonollá et al. (2009), Spain [18]	Yes	No	Yes	Yes	Yes	Yes	Yes	Yes	Yes	Unclear	Ø
Galan et al. (2010), France [14]	Yes	No	Yes	Yes	Yes	Yes	Yes	Yes	Yes	Yes	Ø
Szabo et al. (2012), France [17]	Yes	No	Yes	Yes	Yes	Yes	Yes	Yes	Yes	Yes	Ø
Earnest et al. (2012), UK [19]	Yes	Yes	Yes	Yes	Yes	Yes	Yes	Yes	Yes	Unclear	+

De Natale C et al. (2012), Italy [4]	Yes	No	Yes	Yes	Yes	Yes	Yes	Yes	Yes	No	Ø
Blacher et al. (2013), France [6]	Yes	No	Yes	Yes	Yes	Yes	Yes	Yes	Yes	Yes	Ø
Garaiova et al. (2013), Slovakia [5]	Yes	No	Yes	Yes	Unclear	Yes	Yes	Yes	Yes	No	Ø
Huang et al. (2015), China [3]	Yes	No	No	Yes	Yes	Yes	Yes	Yes	Yes	Yes	Ø

* Negative (−): if most (≥6) of the answers to the above validity questions are 'No', the report should be designated with a minus (−) symbol on the Evidence Worksheet. Neutral (Ø): if the answers to validity criteria questions 2, 3, 6, and 7 do not indicate that the study is exceptionally strong, the report should be designated with a neutral (∅) symbol on the Evidence Worksheet. Positive (+): if most (six or more) of the answers to the above validity questions are 'Yes' (including criteria 2, 3, 6 and 7), the report should be designated with a plus symbol (+) on the Evidence Worksheet.
